# Inhibitory Plasticity Permits the Recruitment of CA2 Pyramidal Neurons by CA3^1^,^2^,^3^

**DOI:** 10.1523/ENEURO.0049-15.2015

**Published:** 2015-07-27

**Authors:** Kaoutsar Nasrallah, Rebecca A. Piskorowski, Vivien Chevaleyre

**Affiliations:** Team Synaptic Plasticity and Neural Networks, FR3636, Centre National de la Recherche Scientifique, Unité Mixte de Recherche 8118, Université Paris Descartes, Sorbonne Paris Cité, 75006 Paris, France

**Keywords:** area CA2, δ opioid receptor, disinhibition, hippocampus, interneuron, long-term depression

## Abstract

Area CA2 is emerging as an important region for hippocampal memory formation. However, how CA2 pyramidal neurons (PNs) are engaged by intrahippocampal inputs remains unclear. Excitatory transmission between CA3 and CA2 is strongly inhibited and is not plastic. We show in mice that different patterns of activity can in fact increase the excitatory drive between CA3 and CA2. We provide evidence that this effect is mediated by a long-term depression at inhibitory synapses (iLTD), as it is evoked by the same protocols and shares the same pharmacology. In addition, we show that the net excitatory drive of distal inputs is also increased after iLTD induction. The disinhibitory increase in excitatory drive is sufficient to allow CA3 inputs to evoke action potential firing in CA2 PNs. Thus, these data reveal that the output of CA2 PNs can be gated by the unique activity-dependent plasticity of inhibitory neurons in area CA2.

## Significance Statement

Long overlooked, recent work has demonstrated that area CA2 of the hippocampus is a critical region for social memory and aggression. How area CA2 integrates into the hippocampal circuit is not understood. While CA2 pyramidal neurons (PNs) receive excitatory input from CA3 PNs, these inputs cannot drive action potentials in CA2 PNs because of a large feedforward inhibition. Furthermore, CA2 PNs do not express long-term potentiation, so it is unclear whether CA2 PNs can be engaged by CA3. We demonstrate that a unique activity-dependent plasticity of CA2 interneurons can increase the excitatory/inhibitory balance between CA3 and CA2 PNs, and allow the recruitment of CA2 PNs. Therefore, our results reveal a mechanism by which CA2 PNs can be engaged by CA3.

## Introduction

Recent findings have revealed that hippocampal area CA2 plays an important role in learning and memory. Area CA2 has been shown to be necessary for social memory formation (Hitti and Siegelbaum, 2014; Stevenson and Caldwell, 2014), and may act to detect discrepancies between stored memories and current sensory information (Wintzer et al., 2014). It has recently been found that the receptive fields of CA2 pyramidal neurons (PNs) change rapidly with time, indicating that this region is playing a role in hippocampal learning that may be separate from spatial orientation (Mankin et al., 2015). Neurons in area CA2 seem poised to modulate hippocampal activity, given the striking number of receptors that are enriched or uniquely expressed in this area (Jones and McHugh, 2011). A clearer understanding of the connectivity in area CA2 has recently evolved (Chevaleyre and Siegelbaum, 2010; Cui et al., 2013; Rowland et al., 2013; Kohara et al., 2014); however, a better understanding of the plasticity of these connections is critical for understanding how this region contributes to hippocampal learning.

Axons from CA3 PNs [i.e. the Schaffer collaterals (SCs)] connect both CA2 and CA1 PNs. However, unlike the well described SC–CA1 synapses, the SC–CA2 connection does not display activity-dependent long-term potentiation (LTP; Zhao et al., 2007; Chevaleyre and Siegelbaum, 2010) due to the unique expression of calcium-buffering proteins (Simons et al., 2009) and of the regulator of G-protein signaling RGS14 (Lee et al., 2010). Furthermore, there is a higher density of interneurons in area CA2 compared with CA1 (Benes et al., 1998; Andrioli et al., 2007; Piskorowski and Chevaleyre, 2013; Botcher et al., 2014). While inhibition is known to control the strength and plasticity of excitatory transmission in the hippocampus (Chevaleyre and Piskorowski, 2014), the control exerted by inhibition in area CA2 is extensive. When inhibitory transmission is intact, electrical (Chevaleyre and Siegelbaum, 2010) or selective optogenetic (Kohara et al., 2014) stimulation of the SC inputs results in a very small depolarizing postsynaptic potential (PSP) comprising both excitatory and inhibitory postsynaptic potential (EPSPs and IPSPs). The depolarizing EPSP is abruptly truncated by a large hyperpolarizing component due to inhibitory currents from feedforward inhibition. This very large feedforward inhibition completely prevents CA3 excitatory inputs from evoking action potentials (APs) in CA2 PNs (Chevaleyre and Siegelbaum, 2010). After pharmacological block of inhibition, the amplitude of the PSP becomes approximately fivefold larger and is sufficient to drive action potentials in CA2 PNs (Chevaleyre and Siegelbaum, 2010; Piskorowski and Chevaleyre, 2013).

Inhibitory transmission from parvalbumin-expressing (PV^+^) interneurons in area CA2 undergoes a long-term depression at inhibitory synapses (iLTD) mediated by δ opioid receptor (DOR) activation (Piskorowski and Chevaleyre, 2013). However, it is unknown whether the synapses that undergo iLTD are playing any role in controlling the net strength of SC–CA2 transmission. We show that the balance between excitation and inhibition between CA3 and CA2 is persistently altered after the induction of DOR-mediated iLTD. Furthermore, the induction of iLTD by SC stimulation also permits a net increase in the excitation/inhibition ratio at distal CA2 inputs. Finally, this disinhibitory mechanism is sufficient to allow SC inputs to drive AP firing in CA2 PNs, thereby engaging CA2 in the trisynaptic circuit.

## Materials and Methods

### Slice preparation

All animal procedures were performed in accordance with the regulations of the animal care committee of the Université Paris Descartes. The 400 µm transverse hippocampal slices were prepared from 6- to 9-week-old C57BL6 male mice. Animals were killed under anesthesia with isoflurane. Hippocampi were removed, placed upright into an agar mold, and cut with a vibratome (VT1200S, Leica) in an ice-cold extracellular solution containing the following (in mm): 10 NaCl, 195 sucrose, 2.5 KCl, 15 glucose, 26 NaHCO_3_, 1.25 NaH_2_PO_4_, 1 CaCl_2_, and 2 MgCl_2_. A cut was made between CA3 and CA2 with a scalpel in some of the slices before being transferred to 30°C ACSF (in mm: 125 NaCl, 2.5 KCl, 10 glucose, 26 NaHCO_3_, 1.25 NaH_2_PO_4_, 2 Na Pyruvate, 2 CaCl_2_ and 1 MgCl_2_) for 30 min and kept at room temperature for at least 1.5 h before recording. All experiments were performed at 33°C. Cutting and recording solutions were both saturated with 95% O_2_ and 5% CO_2_, pH 7.4.

### Electrophysiological recordings and analysis

Field recordings of PSPs were performed in current-clamp mode with a recording patch pipette (3–5 MΩ) containing 1 m NaCl and positioned in the middle of stratum radiatum (SR) or stratum pyramidale in CA1 or CA2. Whole-cell recordings were obtained from CA2 PNs in current-clamp mode held at −73 mV with a patch pipette (3–5 MΩ) containing the following (in mm): 135 K methyl sulfate, 5 KCl, 0.1 EGTA-Na, 10 HEPES, 2 NaCl, 5 ATP, 0.4 GTP, 10 phosphocreatine, and 5 µm biocytin, pH 7.2 (280–290 mOsm). Inhibitory currents were recorded with pipette solution containing 135 Cs methyl sulfate instead of K methyl sulfate. The liquid junction potential was ∼1.2 mV, and membrane potentials were corrected for this junction potential. Some recordings were performed with the perforated patch technique. For these experiments, 75 μg/ml gramicidin was added to the intracellular solution along with an additional 4 mm calcium to ensure that recordings were acquired in perforated configuration only. Series resistance (typically, 12–18 MΩ for whole-cell recordings and 65.5 ± 2 MΩ for perforated patch recordings) was monitored throughout each experiment; cells with a >15% change in series resistance were excluded from analysis. Synaptic potentials were evoked by monopolar stimulation with a patch pipette filled with ACSF and positioned in the middle of CA1 SR or stratum lacunosum-moleculare (SLM). When evoking PSPs in both SR and SLM in the same experiment, we tested the independence of the inputs prior to the experiment. Using the same stimulation intensity used for the experiment, we compared the PSP amplitude evoked by SR stimulation with the PSP amplitude evoked by SR stimulation 100 ms after SLM stimulation. We also performed the reverse measurement, comparing the PSP amplitude evoked by SLM stimulation to a PSP evoked with SLM stimulation after SR stimulation by 100 ms. The stimulation of the SR and SLM inputs was considered to be independent if a preceding evoked PSP in the separate pathway had no effect on the amplitude of the second evoked PSP. When axons of CA2 pyramidal neurons were directly recruited by the stimulation pipette, as observed with a back-propagating AP in the recorded CA2 neuron, the stimulating pipette was moved until the direct activation of the axon disappeared.

High-frequency stimulation (HFS; 100 pulses at 100 Hz repeated twice), 10 Hz stimulation (100 pulses at 10 Hz repeated twice), or DPDPE ([D-Pen^2,5^]Enkephalin) was applied after a stable baseline of 15–20 min duration. Before beginning whole-cell experiments, we identified the CA2 PNs by somatic location and size. Furthermore, the cell type was confirmed by several electrophysiological properties (input resistance, membrane capacitance, resting membrane potential, sag amplitude, action potential amplitude, and duration). For several experiments, particularly when Cs^+^ was in the pipette solution, the slices were fixed after the recording with 4% paraformaldehyde, and the neurons were identified by biocytin-streptavidin labeling and immunohistochemistry to label the CA2-specific protein RGS-14.

The amplitudes of the PSPs were normalized to the baseline amplitude. The magnitude of plasticity was estimated by comparing averaged responses at 30–40 min for whole-cell recordings and at 50–60 min for extracellular recordings after the induction protocol with baseline-averaged responses 0–10 min before the induction protocol. All drugs were bath applied after dilution into the external solution from concentrated stock solutions. We used Axograph X software for data acquisition, and Origin Pro for data analysis. A Student’s *t* test was performed for statistical comparisons, and results are reported as the mean ± SEM.

## Results

**Figure 1:**
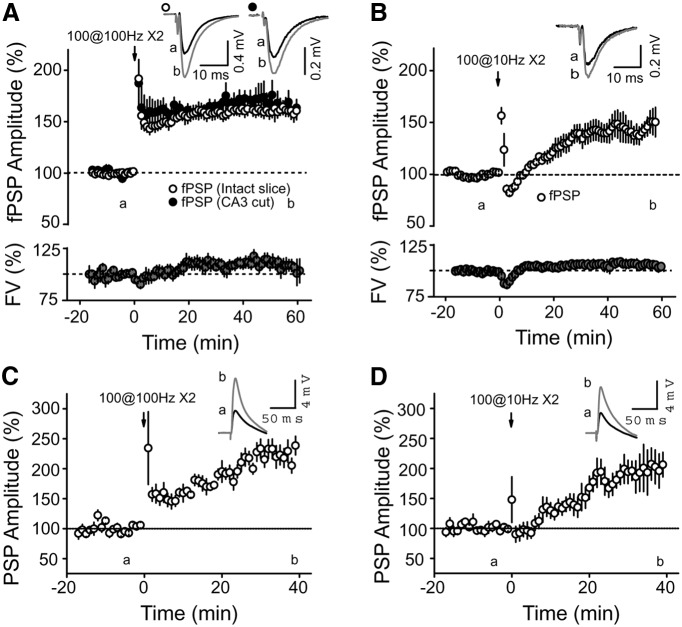
HFS and 10 Hz stimulation induce a long-term increase of SC–CA2 PSP amplitude. ***A–D***, Time course of the average normalized PSP amplitude obtained by extracellular recording (***A***, ***B***) in CA2 SR or whole-cell current-clamp recording (***C***, ***D***) of CA2 PNs, in response to SC stimulation. Both an HFS protocol (two sets of 100 pulses at 100 Hz; ***A***: *p* < 0.00001, *n* = 10; ***C***: *p* = 0.00018, *n* = 9) and a 10 Hz protocol (two sets of 100 pulses at 10 Hz; ***B***: *p* = 0.0009, *n* = 8; ***D***: *p* = 0.01596, *n* = 6) induce a long-term increase in the SC PSP amplitude in CA2. The fiber volley (FV; a measure of the number of axons firing an action potential) was not significantly increased after HFS (***A***; *p* = 0.06) or 10 Hz stimulation (***B***; *p* = 0.19). ***A*** also shows that making a cut between CA3 and CA2 does not affect the magnitude of the potentiation evoked by HFS (*p* = 0.59 with uncut slices, *n* = 5). Top right-hand corner in all panels, Averaged PSP traces of a representative experiment corresponding to time points before (a) and 60 min after (b; ***A***, ***B***) or 40 min after (b; ***C***, ***D***) the stimulation protocol. Error bars indicate the SEM in all panels.

Inhibition in area CA2 has been shown to play a very prominent role in controlling the size of the depolarizing component of the compound EPSP–IPSP after stimulation of the SC (Piskorowski and Chevaleyre, 2013). Furthermore, inhibition has been shown to be highly plastic, undergoing an iLTD mediated by DORs (Piskorowski and Chevaleyre, 2013). We asked whether this plasticity of inhibitory transmission might be sufficient to modulate the level of excitatory drive at SC–CA2 synapses. To address this question, we first recorded extracellular field PSPs (fPSPs) in CA2 SR in response to electrical stimulation of SC fibers. These fPSPs are a compound readout of both the local EPSPs and IPSPs. After a stable baseline period, we applied either an HFS protocol (100 pulses at 100 Hz repeated twice) or a 10 Hz protocol (100 pulses at 10 Hz repeated twice). These two protocols efficiently induce iLTD of inhibitory inputs in area CA2 (Piskorowski and Chevaleyre, 2013). We found that both the HFS and 10 Hz protocol evoked a lasting increase in the amplitude of the compound fPSP [with 100 Hz stimulation: 160.5 ± 4.2% of fPSP amplitude, *p* < 0.00001, *n* = 10 (Fig. 1*A*); with 10 Hz stimulation: 144.8 ± 9.9% of fPSP amplitude, *p* = 0.0034, *n* = 8 (Fig. 1*B*)]. We also observed a significant but smaller increase in the compound fPSP when measuring the slope (136.2 ± 6.4% of fPSP slope, *p* = 0.0009, *n* = 8). We expected a smaller change in the fPSP when measuring the slope, as most of the inhibition evoked by the stimulation is recruited by the SC. The extrasynaptic delay of this feedforward inhibition onto CA2 PNs (compared with the direct SC transmission) will result in a larger control of the peak rather than of the slope of the fPSP. Therefore, the amplitude of the PSP was used for the analysis of the subsequent experiments.

To address whether a change in the excitability of CA3 PNs was responsible for the increase in the fEPSP in CA2, we measured the amplitude of the fiber volley (a measure of the number of axons firing an action potential). As shown in Figure 1, *A* and *B*, we did not detect any significant increase in the fiber volley amplitude after HFS (112.0 ± 5.3%, *p* = 0.06) or after 10 Hz stimulation (105.8 ± 4.1, *p* = 0.19). In addition, we also applied HFS in slices that had a detached CA3. In this condition, we found a similar increase of the compound fPSP amplitude [Fig. 1*A*; 165 ± 7.8% of fPSP amplitude, *n* = 5, *p* = 0.0011 (*p* = 0.59 with uncut slices)]. These data indicate that the long-term increase in the amplitude of the compound fPSP in area CA2 is not due to recurrent activation of CA3 neurons.

We also tested the effect of HFS and 10 Hz stimulation in whole-cell current-clamp recordings of CA2 PNs to ascertain that the increased amplitude of the fPSP was a result of an increased excitatory/inhibitory ratio onto CA2 PNs. We found that both the HFS and 10 Hz protocols triggered a large increase in the PSP recorded in this condition (Fig. 1 *C*,*D*; 223.4 ± 14.1% after HFS, *n* = 9; 195.5 ± 26.3% after 10 Hz stimulation, *n* = 6). The larger increase in PSP amplitude observed in whole-cell recording compared to extracellular recording is not surprising as DORs have been reported to be expressed in interneurons that target PN soma and proximal dendrites (Erbs et al., 2012). A potential iLTD at somatic inputs will likely have a minor contribution in regulating the size of the fPSP amplitude recorded in the dendritic area, but will have a large impact on PSP amplitude measured by whole-cell recordings. Consistent with this idea, we found that fPSPs monitored in the somatic region show a larger increase after HFS compared with fPSP monitored simultaneously in the dendritic area (fPSP in soma, 222.3 ± 28.4%; fPSP in dendrite, 164.3 ± 7.1%; *n* = 5, *p* = 0.047). Thus, our data show that different patterns of activity can increase the excitatory drive between CA3 and CA2.

### Inhibitory transmission is mandatory for the HFS-induced long-term increase of the SC–CA2 PSP

We hypothesize that the increase in the amplitude of the PSP we measured is the consequence of the previously described DOR-mediated iLTD at inhibitory synapses (Piskorowski and Chevaleyre, 2013). That is, this increase in amplitude is due to a disinhibitory mechanism, and does not occur as the result of a potentiation of excitatory transmission. If this prediction is true, then the lasting increase in the compound PSP amplitude can only occur if inhibitory transmission is left intact.

**Figure 2:**
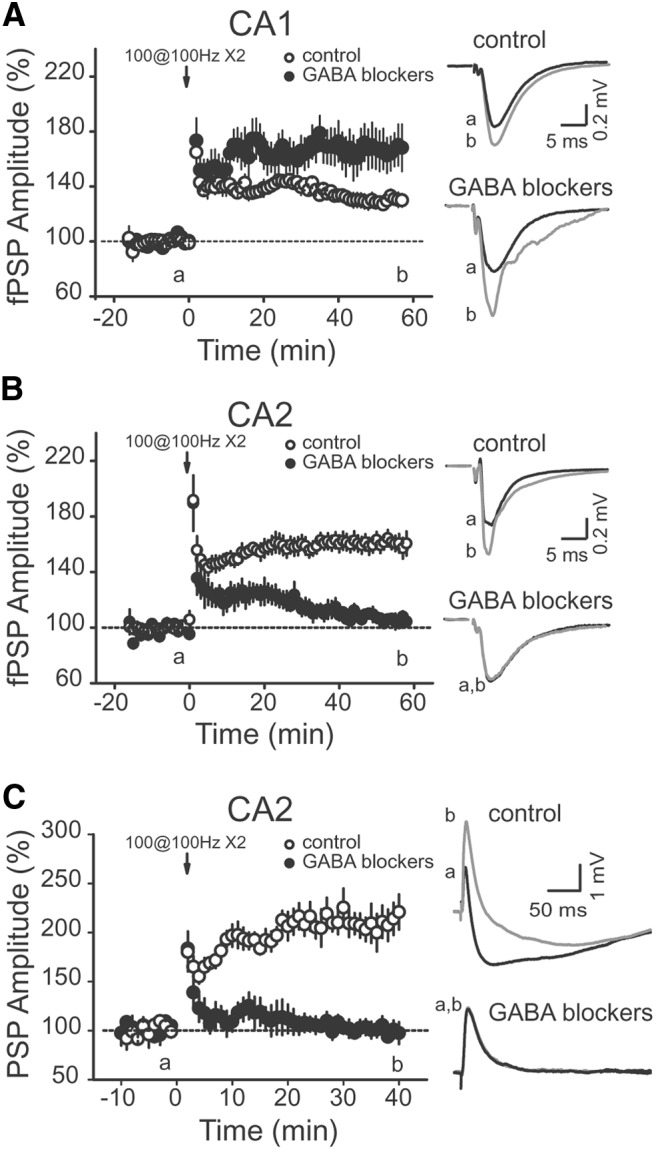
The HFS-induced long-term potentiation of the PSP in CA2 is dependent on GABAergic transmission. ***A***, Time course of average normalized fPSP amplitude recorded in CA1 SR in response to SC stimulation, showing that the HFS-induced increase in PSP amplitude in control conditions (open circles, *p* = 0.0020, *n* = 5) was facilitated in the continuous presence of the GABA_A_ and GABA_B_ receptor antagonists 1 μm SR 95531 and 2 μm CGP 55845 (filled circles, *p* = 0.0017, *n* = 8). ***B***, ***C***, In CA2, HFS does not trigger long-lasting increase in the SC PSP amplitude in the continuous presence of GABA receptor antagonists (filled circles; ***B***, extracellular recordings, *p* = 0.09, *n* = 10; ***C***, whole-cell recording, *p* = 0.6997, *n* = 10), but evokes a large and lasting increase in the PSP amplitude in control experiments (open circles; ***B***, extracellular recordings, *p* < 0.00001, *n* = 10; ***C***, whole-cell recording, *p* < 0.00001, *n* = 10). In all panels, averaged PSP traces corresponding to the time points before (a) and after (b) HFS are shown on the right. Error bars indicate the SEM in all panels.

To test this hypothesis, we recorded extracellular compound fPSPs in areas CA1 and CA2 in response to HFS of SC inputs either in the presence or absence of GABA_A_ and GABA_B_ receptor antagonists (1 µm SR 95531 and 2 µm CGP 55845). In area CA1, the HFS induced a small increase in the fPSP amplitude when inhibition was intact (Fig. 2*A*; 128.2 ± 4.1% of fPSP amplitude, *p* = 0.0020, *n* = 5). As expected, this increase was significantly enhanced when the recordings were performed in the continuous presence of GABA receptor antagonists [Fig. 2*A*; 167.9 ± 13.4%, *p* = 0.0017 (*p* = 0.0447 with intact inhibition), *n* = 8]. In area CA2, a robust and lasting increase of the fPSP was induced with inhibition intact (Fig. 2*B*; 160.5 ± 4.2% of PSP basal amplitude, *p* < 0.00001, *n* = 10). Furthermore, unlike what was observed in area CA1, the HFS did not evoke any lasting change in the fPSP amplitude when inhibitory transmission was blocked [Fig. 2*B*; 104.9 ± 2.3% of EPSP amplitude, *p* = 0.09 (*p* < 0.0001 with intact inhibition), *n* = 10].

We also performed the same experiment using whole-cell current-clamp recordings of CA2 PNs. Consistent with the extracellular recordings, the HFS induced an increase in the amplitude of the depolarizing response when inhibition was intact (Fig. 2*C*; 207.4 ± 12.6% of PSP amplitude, *p* < 0.00001, *n* = 10), but not in the presence of GABA receptor antagonists [Fig. 2*C*; 103.1 ± 5.7% of EPSP amplitude, *p* = 0.6997 (*p* = 0.000006 with intact inhibition), *n* = 7]. Altogether, these results show that in area CA2, intact inhibitory transmission is a requirement for the long-term increase of the compound PSP amplitude.

**Figure 3:**
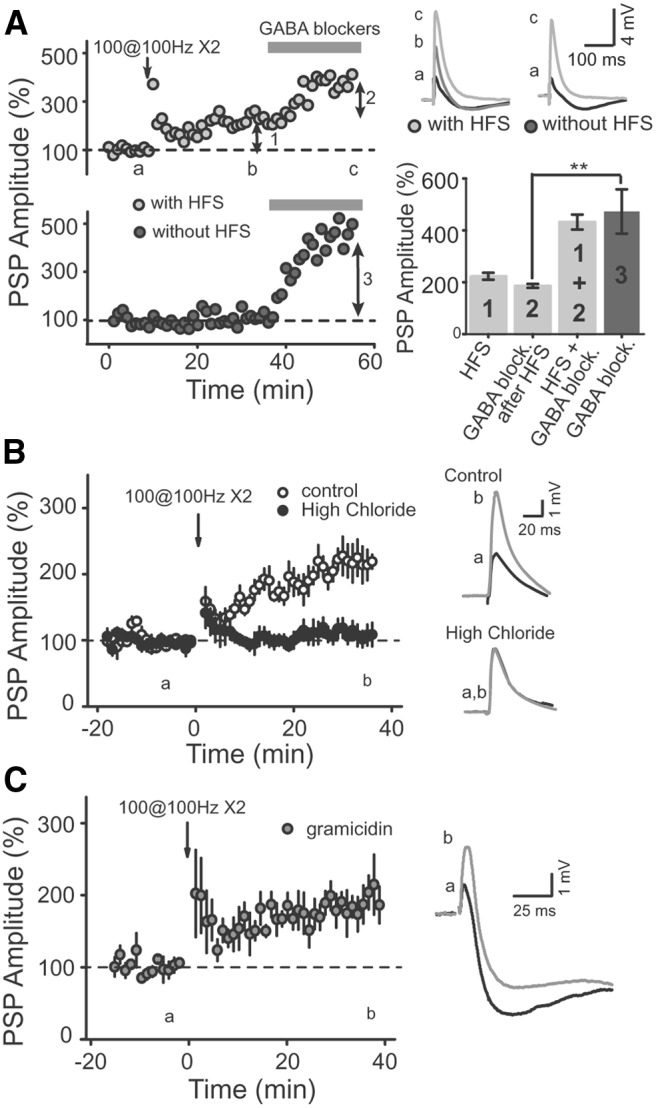
HFS induces a long-term increase of the PSP amplitude in CA2 via a disinhibition mechanism. ***A***, Two representative examples of the normalized PSP time course from CA2 PN whole-cell recordings illustrating how an HFS partially occludes the effect of GABA_A_ and GABA_B_ receptor blocker application on PSP amplitude. Top right, PSP traces corresponding to the time points before (a), after HFS (b), and after the application of GABA receptor blockers (c) with or without HFS. Bottom right, Summary histograms showing the percentage increase in the PSP amplitude induced by HFS applied alone (1), GABA receptor blocker application after HFS (2), HFS plus GABA blockers (1 + 2), and GABA blockers applied without previous HFS (3). ***B***, Normalized CA2 PN SC PSPs recorded with either 7 mm (open circles) or 16 mm (closed circles) [Cl−] in the pipette solution. An HFS (arrow at time 0) fails to induce a long-lasting increase in PSP amplitude when a high concentration of chloride is used in the pipette solution (filled circled, *p* = 0.4103, *n* = 5) but evokes normal long-term potentiation in control experiments (open circles, *p* = 0.0047, *n* = 5). ***C***, Summary graph of experiments performed using the gramicidin-perforated patch-recording configuration. HFS triggers an increase in PSP amplitude (*p* = 0.008, *n* = 5) similar to the one observed using whole-cell recording configuration.

If the increase in the PSP after HFS indeed results from a decrease in inhibition, then it is possible to make the following prediction: pharmacological block of inhibition should increase the amplitude of the PSP, and part of this increase should be occluded by a previous HFS. In order to test this, we performed current-clamp recordings of CA2 PNs and applied GABA receptor blockers after an HFS of SC inputs, or in control experiments with no tetanus stimulation. We found that when the GABA receptor blockers were applied without the HFS, the PSP amplitude was strongly increased (Fig. 3*A*; 472.8 ± 85.5% of the PSP amplitude, *p* = 0.0026, *n* = 6), confirming that GABAergic transmission exerts a strong negative control on the PSP amplitude in CA2 PNs (Piskorowski and Chevaleyre, 2013). When the GABA receptor blockers were applied after the HFS, the increase in the PSP amplitude was smaller than the effect of the blockers applied without the HFS [Fig. 3*A*; 185.8 ± 7.8% of increase of the PSP amplitude, *p* < 0.00001 (*p* = 0.0074 compared with blockers without HFS), *n* = 6]. However, the final PSP amplitude after the HFS and GABA blocker application was identical to the amplitude of the PSP after GABA blocker application alone (Fig. 3*A*; *p* = 0.7, *n* = 6). Altogether, these results indicate that the HFS-induced increase of the PSP amplitude is mediated by a decrease in GABAergic transmission.

It was previously reported that the SC–CA2 synapse does not express LTP when inhibition is pharmacologically blocked, but also when inhibition is kept intact (Zhao et al., 2007). One potential explanation for the apparent discrepancy with our results could be the difference in chloride used in our internal solution and that of the previous study. The intracellular [Cl^−^] of our pipette solution is 7 mm, making the reversal potential for Cl− (E_Cl_) of −77 mV. The previous study (Zhao et al., 2007) used 16 mm, with an E_Cl_ of −55 mV, which is more depolarized than the resting membrane potential of the cell. With the higher [Cl^−^] in the intracellular solution, a decrease in GABAergic transmission would likely not increase the compound PSP amplitude. To test whether the difference in E_Cl_ could explain the discrepancy between our results and those of Zhao et al. (2007), we performed whole-cell current-clamp recordings of CA2 PNs with an internal solution containing 16 mm Cl^−^. We found that an HFS did not evoke an increase in the amplitude of the PSP in CA2 PNs when using a high concentration of chloride in the pipette, even with intact GABAergic transmission [Fig. 3*B*; 111.0 ± 16.9% of PSP basal amplitude, *p* = 0.4103 (*p* = 0.006 with control slices) *n* = 5]. Finally, in order to directly monitor the compound EPSP/IPSP in pyramidal neurons without affecting intracellular [Cl^−^], we performed perforated patch recordings with gramicidin in the pipette. In these conditions, we found that HFS evoked a large increase in the amplitude of the PSP that was not different than the one observed with whole-cell recordings (Fig. 3*C*; 200.8 ± 19.9% of baseline, *n* = 5, *p* = 0.008 with baseline, *p* = 0.7 compared with whole-cell experiments). Altogether, these data strongly indicate that the disinhibitory increase in PSP amplitude can occur during physiological conditions.

### The long-term increase in the CA2 compound PSP amplitude requires the activation of δ opioid receptors

Our results show that the potentiation of the PSP amplitude in area CA2 induced by an HFS results from a decrease in inhibition, rather than from a direct potentiation of excitatory inputs. If iLTD in area CA2 indeed mediates the increase in the compound PSP, both iLTD and the increase in PSP amplitude should share similar pharmacology. Because the induction of iLTD in area CA2 requires the activation of DORs (Piskorowski and Chevaleyre, 2013), we tested whether HFS-mediated increase in PSP amplitude is also dependent on the activation of these receptors.

**Figure 4:**
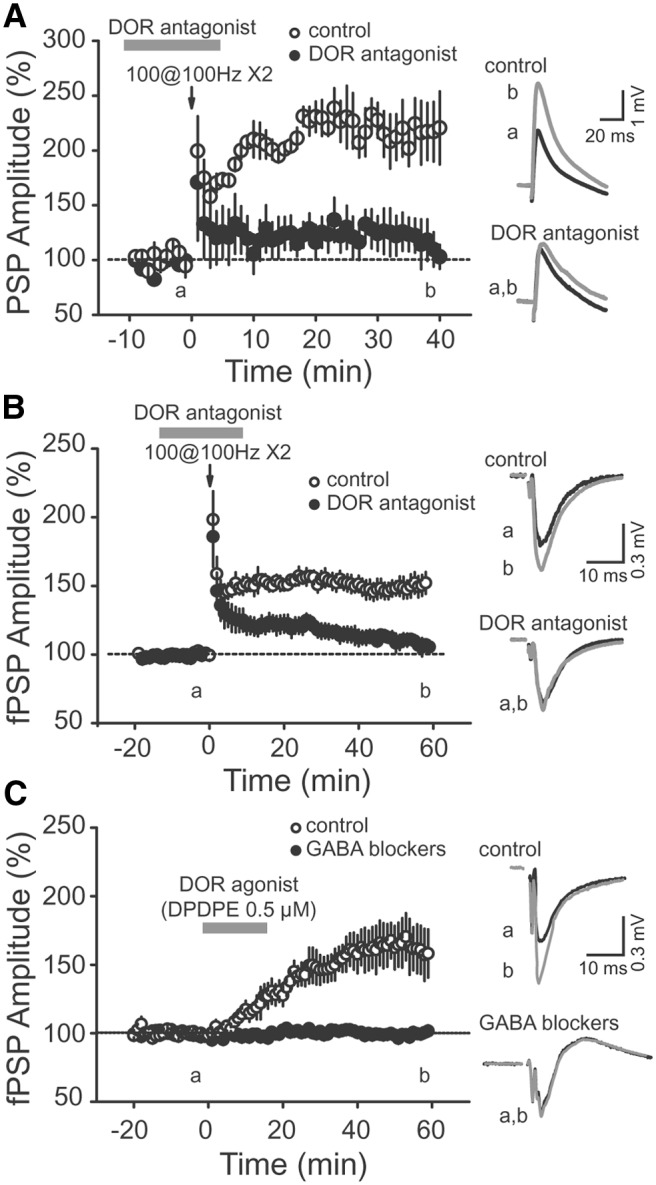
The increase in SC PSP amplitude in CA2 PNs is dependent on DOR activation. ***A***, An HFS does not trigger a long-term increase of the PSP amplitude recorded in CA2 PNs in the presence of 2 μm DOR competitive antagonist ICI 174864 (filled circles, *n* = 7, *p* = 0.135) but induces a normal long-term increase in the PSP magnitude in interleaved control experiments (open circles, *p* = 0.005, *n* = 5). ***B***, Time course of normalized fPSP amplitude recorded in CA2 SR showing how the application of a DOR antagonist (ICI 174864 or naltrindole 0.1 μm) during HFS (filled circles, *p* = 0.0822 and *p* = 0.0006 with the absence of ICI 74864, *n* = 8) prevented the induction of a lasting increase in the fPSP amplitude that was observed in the absence of drug application (open circle). Averaged PSP traces corresponding to the time points before (a) and after (b) HFS performed in control conditions (top) or in the presence of ICI 174864 (bottom) are shown on the right. ***C***, Application of 0.5 μm DOR-selective agonist (DPDPE) is sufficient to induce a long-lasting increase in the fPSP amplitude recorded in SR of CA2 in the absence (open circles, *p* = 0.02, *n* = 5) but not in the presence (filled circles, *p* = 0.55679, *n* = 6) of GABA_A_ and GABA_B_ receptor blockers. Right, Example fPSP traces corresponding to the time points before (a) and after (b) the application of DPDPE in the absence of (top) or in the continuous presence of (bottom) the GABA_A_ and GABAB receptor blockers. Error bars indicate the SEM in all panels.

First, we performed extracellular and whole-cell current-clamp recordings of CA2 PNs in response to SC stimulation and applied an HFS in the presence of a DOR competitive antagonist, ICI 74864. We found that the HFS did not significantly change the CA2 PN PSP amplitude if DORs were not activated during the tetanus stimulation (Fig. 4*A*; ICI 74864: 119.6 ± 12.9% of the PSP amplitude, *n* = 7, *p* = 0.135; interleaved control: 215.5 ± 20.6% of the PSP amplitude, *p* = 0.005, *n* = 5). Similarly, using extracellular recordings, we found no lasting change in the amplitude of the fPSPs after HFS in the presence of DOR antagonists ICI 74864 or naltrindole [Fig. 4*B*; 108.7±4.6% of the fPSP basal amplitude, *p* = 0.0822 (*p* = 0.0006 with the absence of DOR antagonist), *n* = 8]. For these experiments, we used two structurally distinct DOR antagonists (ICI 74864 and naltrindole). Because the increase in PSP amplitude was blocked with both antagonists, data were pooled.

Together, these data show that DOR activation is needed for induction of the lasting increase in CA2 PN PSP amplitude. We then asked whether the activation of DORs would be sufficient to mediate this plasticity. To address this question, we performed extracellular recordings in the SR of area CA2 and applied the specific DOR agonist DPDPE for 15 min. We found that the application of DPDPE induced a long-term increase in the fPSP amplitude (Fig. 4*C*; 162.2 ± 17.7% of baseline, *p* = 0.02, *n* = 5). To make sure that this long-term potentiation is due to a DOR-dependent decrease in inhibition and not to a potential direct increase in SC–CA2 excitatory transmission, we performed the same experiment in the presence of GABA receptor blockers. We found that when inhibitory transmission was blocked, DOR activation did not induce any lasting increase in the PSP amplitude at SC–CA2 synapses (Fig. 4*C*; 99.8 ± 4.2% of baseline, *p* = 0.71, *n* = 6). Altogether, these data show that DOR activation is necessary and sufficient to trigger a lasting increase in the amplitude of the PSP between SC and CA2 PNs.

### iLTD evoked by SC input stimulation also alters the excitatory/inhibitory balance at distal inputs of CA2 PNs

**Figure 5:**
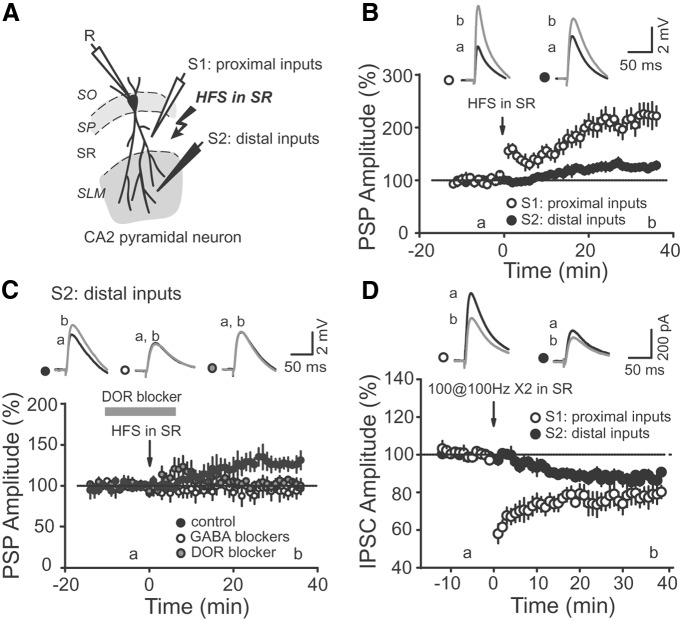
Stimulation in SR induces a heterosynaptic iLTD and increases distal excitatory drive onto CA2 PNs. ***A***, Cartoon illustrating the arrangement of the stimulating recording electrodes in SR and SLM. ***B***, Average PSP amplitudes of SR (open circles) and SLM (closed circles) inputs after HFS stimulation in SR. Note that both SR and SLM inputs are potentiated after the HFS (*p* = 0.00035 for SR inputs, *p* = 0.0017 for SLM inputs, *n* = 10), but only SR inputs show a rapid post-tetanic increase in amplitude. Top, Averaged PSP traces corresponding to the time points before (a) and after (b) HFS. ***C***, The increase in distally evoked PSP after stimulation in SR was blocked by GABA_A_ and GABA_B_ receptor blockers (open circles, *p* = 0.52, *n* = 8) and by the DOR antagonist naltrindol (gray circles, *p* = 0.31, *n* = 6). ***D***, Average amplitude of IPSCs evoked by stimulation in SR and SLM after HFS in SR. Note that both inputs express an inhibitory LTD after HFS in SR (*p* = 0.006 for SR inputs; *p* = 0.003 for SLM inputs, *n* = 6).

CA2 PNs are strongly activated by distal inputs in SLM. Thus, we wondered whether the stimulation of proximal SC inputs, via a disinhibitory mechanism, could also influence distal inputs in SLM. We used two stimulating electrodes to evoke PSPs in SC and SLM inputs, as diagrammed in Figure 5*A*, and checked for the independence of the two pathways before starting experiments (see Materials and Methods). We found that tetanic stimulation in SR not only led to a large increase in the PSP amplitude of SC inputs, but also increased the amplitude of PSPs from SLM inputs (Fig. 5*B*; 125.9 ± 5.9% of baseline, *p* = 0.0017 with baseline, *n* = 10). In contrast to SC inputs, SLM inputs have been described to express an LTP that is independent of inhibitory transmission (Chevaleyre and Siegelbaum, 2010). Thus, we wondered whether our observed increase in SLM PSP is a result of a direct LTP at these inputs or from a disinhibitory mechanism similar to what we have found at SC–CA2 inputs. We repeated the experiment in the presence of GABA receptor blockers and found that the tetanic stimulation in SR did not result in any increase of SLM-evoked PSPs (Fig. 5*C*; 94.6 ± 7.9% of baseline, *p* = 0.52 with baseline, *n* = 8), which is consistent with a disinhibitory mechanism. We then tested whether this disinhibitory mechanism at the SLM pathway also depends on DOR activation by applying the SR tetanus stimulation in the presence of naltrindole. We found that the increase in the PSP amplitude of SLM input was completely blocked under these conditions (Fig. 5*C*; 103.9 ± 3.4% of baseline, *p* = 0.31 with baseline, *n* = 6).

We postulate that this increase in the strength of SLM inputs is a result of the same DOR-dependent disinhibition that increases the strength of SC inputs. To test this hypothesis, we directly monitored IPSCs evoked by stimulation in SR and SLM. We found that tetanus stimulation in SR evokes a lasting and significant iLTD in both SC and SLM inputs into CA2 PNs (Fig. 5*D*; 77.7 ± 4.8% of baseline, *p* = 0.005 for SR IPSCs; 87.0 ± 2.5% of baseline, *p* = 0.003 for SLM IPSCs; *n* = 6).

### The disinhibition at SC–CA2 allows SC inputs to drive firing in CA2 PNs

The large feedforward inhibition recruited by SC stimulation completely prevents action potential firing in CA2 PNs. However, when inhibition is pharmacologically blocked, SC inputs are able to drive action potential firing in CA2 PNs (Chevaleyre and Siegelbaum, 2010). Therefore, we asked whether the decrease in inhibition after iLTD, and the consequent increase in compound PSP amplitude would sufficiently increase the net excitatory drive between CA3 and CA2 PNs to allow SC to drive firing in CA2 PNs.

**Figure 6:**
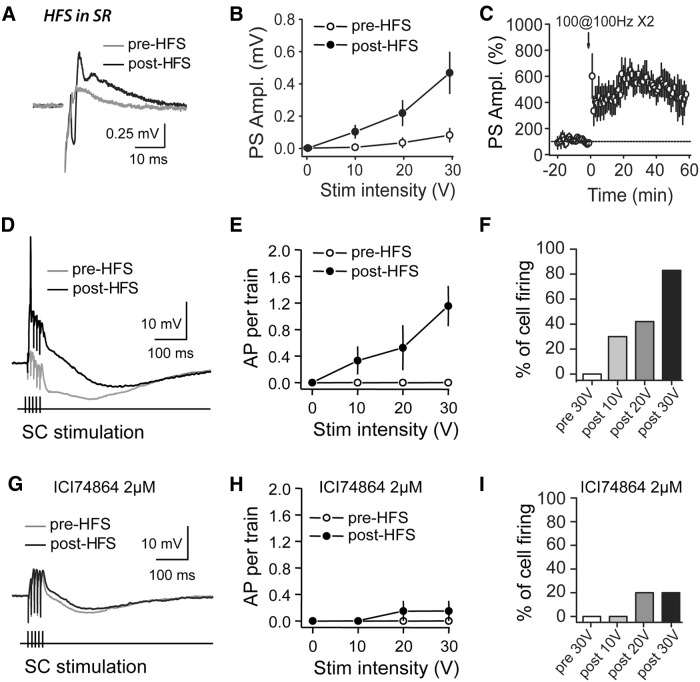
HFS in SR allows CA3 inputs to evoke action potential firing in CA2 PNs. ***A***, Traces of extracellular recordings in the CA2 pyramidal layer in response to a 20 V stimulation of SC inputs, before (gray traces) and after an HFS (black traces), illustrating how HFS induces an increase in the PS amplitude (negative peak). ***B***, Average PS amplitude as a function of stimulation intensity before (open circle) and after (filled circle) HFS (with 20 V stimulation: *p* = 0.01, *n* = 5; with 30 V stimulation: *p* = 0.006, *n* = 5). ***C***, Time course of average normalized PS amplitude recorded in CA2 pyramidal layer in response to a 20 V stimulation of SC inputs, showing a long-lasting increase in PS amplitude after HFS (*p* = 0.01, *n* = 5). ***D***, Traces of whole-cell current-clamp recordings in a CA2 pyramidal cell in response to a train of stimulations (five pulses at 100 Hz) of SC inputs, illustrating how APs can be evoked in CA2 neurons after HFS (black traces) but not before HFS (gray traces). ***E***, Average number of APs per train at different stimulation intensities, showing how a five-pulse train at 100 Hz of the SC inputs does not trigger APs in CA2 PNs before HFS (open circles) but induces APs after HFS (filled circles, with 30 V stimulation: from 0 to 1.15 ± 0.3 APs per train after HFS, *p* = 0.012, *n* = 6). ***F***, An HFS increases the percentage of CA2 PNs firing at least one AP during the train (with 30 V stimulation: from 0% to 80% of cells firing APs). ***G***, Traces of whole-cell current-clamp recordings in a CA2 PN in response to a stimulation train (five pulses at 100 Hz) of SC inputs in the presence of the DOR antagonist ICI 174864 (2 μm), illustrating how the application of ICI 174864 prevents the induction of APs in CA2 PNs after HFS (black traces). ***H***, Average number of APs per train at different stimulation intensities in the presence of the DOR antagonist before HFS (open circles; with 0–30 V stimulations: 0 APs per train) and after HFS (filled circles; with 30 V stimulation: 0.15 ± 0.15 AP per train after HFS, *p* = 0.37, *n* = 5). ***I***, In the presence of ICI 174864, HFS does not induce a large increase in the percentage of CA2 PNs firing at least one AP during the train (with 10 V stimulation: 0% of cells firing APs before and after HFS; with 20 and 30 V stimulation: from 0% to 20% of cells firing APs). Error bars indicate the SEM in all panels.

To test this hypothesis, we monitored the magnitude of the population spike (PS) in the somatic layer of area CA2 before and after HFS. We found that before HFS there was no PS or a PS of very small amplitude that was measurable only at the highest intensity stimulation (30 V). This observation confirms that the stimulation of CA3 SC axons was not capable of driving the firing of PNs in the CA2 region. Interestingly, a large PS was revealed in CA2 after HFS (Fig. 6*A–C*; with 20 V stimulation: PS amplitude increases from 0.035 ± 0.024 to 0.37 ± 0.11 mV after HFS, *p* = 0.01, *n* = 5; with 30 V stimulation: PS amplitude increases from 0.09 ± 0.04 to 0.59 ± 0.14 mV after HFS, *p* = 0.006, *n* = 5). This result shows that the plasticity induced by the HFS is sufficient to allow CA3 inputs to evoke the firing of cells in CA2.

To determine whether the pyramidal cells in area CA2 experience an increased firing probability after HFS of SC, we performed whole-cell current-clamp recordings of CA2 PNs and measured the probability of AP firing in response to a series of five pulses at 100 Hz at different stimulus intensities before and after HFS. Before HFS, no APs were evoked in CA2 PNs. However, after HFS, CA2 PNs were able to generate APs in response to SC stimulation. Both the number of APs per train and the proportion of cells firing at least one AP during the train were significantly increased after HFS (Fig. 6*D–F*; with 30 V stimulation: from 0 to 1.15 ± 0.3 APs per train after HFS, *p* = 0.012, *n* = 6; and from 0% to 80% of cells firing APs after HFS). To ensure that this increase in firing results from the DOR-mediated plasticity at inhibitory synapses evoked by HFS, we performed the same experiment in the presence of the DOR antagonist ICI 174864 (2 µm). When HFS was applied in the presence of the drug, no significant change in action potential firing was evoked (Fig. 6*G–I*; with 30 V stimulation: from 0 to 0.15 ± 0.15 AP per train after HFS, *p* = 0.37, *n* = 5; and from 0% to 20% of cells firing APs after HFS). These results show that the DOR-dependent disinhibitory increase in PSP is sufficient to allow CA3 inputs to drive AP firing in CA2 PNs.

## Discussion

In this study, we have shown that the net excitatory drive between CA3 and CA2 can be persistently increased in an activity-dependent manner, even though the CA3–CA2 excitatory transmission does not express a direct LTP. Our results show that the increase in the excitatory drive between CA3 and CA2 is dependent on inhibition and results from a DOR-mediated iLTD at inhibitory synapses. Furthermore, we show that this disinhibition also increases the excitatory drive between SLM inputs and CA2. Finally, this plasticity sufficiently increases the net excitatory drive between CA3 and CA2 to allow SC inputs to drive action potential firing in CA2 PNs.

### Activity induces a long-term increase of CA3–CA2 transmission via a disinhibitory mechanism

There are several examples of disinhibitory plasticity in the hippocampus. For instance, a pairing protocol induces a shift in GABA reversal potential, thereby increasing synaptic strength at CA3–CA1 synapses (Ormond and Woodin, 2009). Similarly, a pairing protocol between distal and proximal inputs strongly facilitates CA3–CA1 synapses, an effect that is dependent on a decrease in feedforward inhibition recruited by SC synapses (Basu et al., 2013). In both cases, the decrease in inhibition facilitates a plasticity that can occur independently of a change in inhibition. In our study, the increase in excitatory/inhibitory balance between CA3 and CA2 is entirely dependent on a disinhibition mechanism. Indeed, SC–CA2 excitatory synapses do not express LTP after HFS or other induction protocols (Zhao et al., 2007; Simons et al., 2009; Chevaleyre and Siegelbaum, 2010; Lee et al., 2010). However, we found that HFS or 10 Hz stimulation could trigger a lasting increase in the net excitatory drive between CA3 and CA2. Several experiments indicate that this increase is dependent on GABAergic transmission and results from a disinhibitory mechanism. First, the lasting increase in PSP amplitude was absent when inhibitory transmission was pharmacologically blocked. Second, increasing the reversal potential for Cl^−^ in the recorded cell also prevented induction of this plasticity. Third, the increase in the amplitude of the PSP after the application of GABA receptor blockers was partially occluded by previous HFS. Fourth, the activity-dependent increase in PSP shares the same pharmacology as the iLTD (i.e., a strict dependence on DOR activation). And finally, the application of a DOR agonist, which induces a lasting decrease in inhibition but does not alter isolated excitatory transmission, was sufficient to evoke the lasting increase in PSP amplitude.

Our results are in agreement with those of several studies showing a lack of plasticity at SC–CA2 synapses when inhibition was blocked. Nonetheless, they seem to differ from the results of a previous study (Zhao et al., 2007) in which no plasticity was evoked at SC synapses with inhibitory transmission left intact. We believe that the recording conditions likely account for this apparent discrepancy. While the previous study used a high concentration of Cl^−^ in the recording solution (16 mm), we used 7 mm Cl^−^. In accordance with this idea, we found no increase in PSP amplitude with 16 mm Cl^−^ in the recording pipette. The precise physiological concentration of intracellular Cl^−^ in CA2 PNs is unknown. However, the fact that we observe an increase in PSP amplitude when intracellular [Cl^−^] is not perturbed (using gramicidin perforated patch recordings), and when both intracellular ionic composition and resting membrane potential are not affected (using extracellular recordings), strongly indicates that the disinhibition-mediated increase in PSP amplitude can occur in an intact system. Furthermore, we are confident that the fPSP we recorded in CA2 is not contaminated by PSP in CA1 because we did not observe LTP in CA2 in the presence of GABA blockers, while a large potentiation was observed with simultaneous fPSP recordings in CA1.

### The disinhibitory increase in CA3–CA2 transmission is dependent on DOR

Our results strongly indicate that the DOR-mediated iLTD recently described (Piskorowski and Chevaleyre, 2013) is involved in the disinhibitory increase in PSP between CA3 and CA2. First, the disinhibitory potentiation was abolished in the presence of two different selective antagonists of DORs (ICI 74864 and naltrindole). Second, the direct activation of DORs with the selective agonist DPDPE was sufficient to trigger a lasting increase in the PSP amplitude. Therefore, these data show that DORs are necessary and sufficient to increase the PSP amplitude between CA3 and CA2.

Opioids have long been known to increase excitability in the hippocampus and to facilitate the induction of plasticity at excitatory synapses. For instance, the activation of μ opioid receptors (MORs) and DORs is required for LTP induction at the perforant path–granual cell synapse in the dentate gyrus (Bramham and Sarvey, 1996), and the activation of MORs facilitates LTD induction at the SC–CA1 PN synapse (Wagner et al., 2001). In both cases, the observed increase in excitatory drive is believed to result from a transient decrease in inhibition during the induction protocol, hence facilitating the induction of the plasticity at excitatory inputs.

Our results differ in several ways from the previously reported action of opioids. First, in contrast to the transient effect of DORs on GABAergic transmission in CA1, the activation of DORs was shown to induce a lasting depression of inhibition in area CA2 (Piskorowski and Chevaleyre, 2013). In addition, in contrast to the well described facilitatory action of opioids on plasticity at excitatory synapses, our results show that DOR activation is mandatory for the change in excitatory/inhibitory balance between CA3 and CA2.

Our results show that stimulation in SR increases the synaptic strength of both proximal and distal inputs. The increase in the strength of excitatory transmission was dependent on GABAergic transmission. There have been several reports of heterosynaptic plasticity between proximal and distal inputs onto hippocampal PNs; for example, the potentiation of SC inputs onto CA1 PNs after theta-burst stimulation of distal dendritic inputs (Han and Heinemann, 2013), and the potentiation of SC inputs and depression of distal dendritic inputs after low-frequency stimulation of distal inputs (Wöhrl et al., 2007). To our knowledge, there is no report of a lasting increase in synaptic strength at distal dendritic input that is induced by stimulation in the proximal dendritic region. The simplest explanation for our results is that stimulation in SR evokes iLTD, and both SC and SLM stimulation recruit the depressed inhibitory inputs. In agreement with this idea are the findings that PV^+^ basket cells in area CA2 have dendrites that traverse SR and SLM (Mercer et al., 2012; Tukker et al., 2013). Furthermore, it was recently shown that synaptic transmission from PV^+^ interneurons in area CA2 is depressed during DOR-mediated iLTD (Piskorowski and Chevaleyre, 2013). Together, our results highlight a new mechanism by which activity in SR can enhance the net excitatory drive of synapses in SLM. This interplay is likely to have important consequences on information transfer by extrahippocampal (SLM) and intrahippocampal (SC) inputs onto CA2 PNs.

### Physiological and pathological consequences of the disinhibitory action of DOR in CA2

When inhibition is intact, SC EPSPs are very small and fail to induce AP in CA2 PNs. Our results show that, after the induction of DOR-mediated iLTD, the stimulation of SC inputs can evoke APs in CA2 PNs. This was observed both in whole-cell and extracellular recordings, indicating that it can occur when the resting potential of CA2 PNs is not affected. This result is consistent with those of a previous study (Sekino et al., 1997) using voltage-sensitive dyes to study information propagation through the hippocampus reporting that dentate gyrus stimulation resulted in either a fast propagation of activity between CA3 and CA1, or a slower propagation successively recruiting CA3, CA2, and CA1 PNs. Interestingly, the blockade of GABA receptors allowed the recruitment of CA2 from slices in which the activation of this region was initially not detected. Our results provide a mechanism for the result observed in this previous study and show that the disinhibition-dependent recruitment of CA2 can be evoked in an activity-dependent manner. While HFS (100 Hz) might not be very physiological, the activity-dependent increase in PSP amplitude can also be evoked with a 10 Hz stimulation, a frequency that falls within the range of theta oscillations. Furthermore, it was previously shown that the depression of inhibitory transmission underlying the increase in PSP can be evoked with 100 and 10 Hz stimulation, but also with a theta burst stimulation protocol. Therefore, we think it is likely that physiological patterns of activity will be efficient to trigger the increase in excitatory drive between CA3 and CA2, thus allowing CA2 to be recruited by CA3. We propose that inhibition in area CA2 is acting as a gate to control information flow between CA3 and CA2, and that DOR activation can be considered as one of the keys to opening this gate.

While our results suggest that an activity-dependent decrease in inhibition could be relevant for information transfer by allowing SC inputs to activate CA2 PNs, it is likely that a more global decrease in inhibition in CA2 is detrimental for the hippocampus. For instance, a decrease in the density of PV^+^ interneurons has been reported (Knable et al., 2004) to occur uniquely in area CA2 during schizophrenia. Similarly, the density of PV^+^ cells is considerably reduced in CA2 during epilepsy (Andrioli et al., 2007), and physiological recordings in the hippocampi of human epileptic patients revealed an important decrease in inhibition of (Wittner et al., 2009) or a complete loss of inhibition of CA2 PNs (Williamson and Spencer, 1994). Our results provide a potential explanation for why a persistent decrease in the inhibitory gate in CA2 could alter the trisynaptic circuit during schizophrenia (Benes, 1999), and why CA2 might be the locus of epileptiform activity generation both in rodents (Knowles et al., 1987) and in humans (Wittner et al., 2009).

In summary, our results show that while there is no LTP at the SC–CA2 PN synapse, the excitatory/inhibitory balance can be persistently shifted toward excitation in an activity-dependent manner through a disinhibition mechanism. Interestingly, this disinhibition results in an increase in net excitatory drive at both SC inputs as well as distal dendritic inputs. Furthermore, our results reveal that the decrease in inhibitory transmission sufficiently increases the excitatory drive from SC inputs to allow recruitment of CA2 by CA3 PNs. Thus, our data add complexity to the hippocampal circuitry and reveal how CA2 PNs can be engaged by intrahippocampal inputs after activity.

## Synthesis

The decision was a result of the Reviewing Editor Pablo Castillo and the peer reviewers coming together and discussing their recommendations until a consensus was reached. A fact-based synthesis statement explaining their decision and outlining what is needed to prepare a revision is listed below. The following reviewers agreed to reveal their identity: Chris McBain

Your manuscript has been evaluated by two reviewers who agree on the good quality of the results and overall merit of your study in the context of the hippocampal CA2 area function. Below you will find a synthesis of their comments and online discussion.

This manuscript reports that repetitive stimulation (100 Hz and 10 Hz) in stratum radiatum of CA1 potentiates field EPSPs and mixed PSPs recorded in CA2 pyramidal neurons. While previous studies showed that HFS does not induce LTP in CA2, the authors here make a convincing case that the increase in fEPSPs, mixed PSPs and excitatory drive observed after tetanic stimulation results entirely from a long-lasting decrease in disynaptic inhibitory drive to the CA2 pyramidal cells. Moreover, the authors demonstrate the involvement of delta opioid receptors in this disinhibition. The experiments are well designed, the results are clean and the conclusions are sound.

Here I summarize the most relevant of the reviewers' comments and requests.

1. The authors show that with different intracellular Cl- concentrations the effects of HFS are entirely different. However, given the discrepancy with previous study (Zhao et al), it will be important to know what happens to the intracellular PSP when intracellular Cl- is not perturbed, e.g. using gramicidin perforated patch recordings.
2. It would be useful to see recordings of the GABAergic currents on the CA2 cell before and after LTP, in addition to the predicted lack of decrease if DOR antagonists are applied.
3. Figure 1: include a plot of the fiber volley, which can be easily extracted from the dataset in panels A and B. Although the experiments using a cut between the CA3 and CA2 largely confirm no change in the excitability of the CA3 subfield underlying the change in PSP amplitude, a more accurate way would be to show the fiber volley.
4. Given that 10/100Hz repetitive stimulation in s. radiatum with an electrical stimulating electrode is an unlikely model of how these synapses normally operate, some speculation about when this disynaptic inhibition mechanism may be in play would be of interest.

Some text revisions:

- Avoid calling "LTP" to the potentiation of the fEPSP in CA2 (e.g. figure 3A); it is not LTP as it is understood by most people. Likewise, the first sentence of the discussion should be modified. The authors are not measuring the "net excitatory output of a synapse" anywhere in the paper except where GABARs are blocked, and therefore cannot comment on whether its "output" can be persistently increased in an activity-dependent manner.
- In the Figure 3A histogram, clarify the meaning of the two middle bars in the figure legend.
- Page 7 line 19-20. While the conclusion that the E/I balance is inferred from this data the authors have not demonstrated any change in the inhibitory component. At this point in the manuscript the reviewer is agnostic to the underlying mechanism(s) until a change in the inhibitory component is addressed head on. I would change or eliminate this sentence.
- Introduction, line 1: "important role in learning....."
- Page 5, line 10: define what DPDPE is.
